# Self-rated health, ethnicity and social position in a deprived neighbourhood in Denmark

**DOI:** 10.1186/1475-9276-10-5

**Published:** 2011-01-25

**Authors:** Pernille T Andersen, Carsten K Bak, Susanne Vangsgaard, Unni Dokkedal, Pia V Larsen

**Affiliations:** 1 Unit for Health Promotion Research, Institute of Public Health, University of Southern Denmark, Niels Bohrs Vej 9-10, 6700 Esbjerg, Denmark; 2 Danish Center for Rural Research, Department of Environmental and Business Economics, University of Southern Denmark, Niels Bohrs Vej 9-10, 6700 Esbjerg, Denmark; Health office. Municipality of Fredericia, Gothersgade 20, 7000 Fredericia, Denmark; 3 Unit for Health Promotion Research, Institute of Public Health, University of Southern Denmark, Niels Bohrs Vej 9-10, 6700 Esbjerg, Denmark; 4 Research Unit for General Practice, Institute of Public Health, University of Southern Denmark, J. B. Winsløws Vej 9A, 5000 Odense C, Denmark

## Abstract

**Background:**

In recent years the close connection between SES and differences in health between ethnic groups have been subject to growing interest among researchers, and some studies have found an association between ethnicity and long term illness and poor health. However, there is limited research-based knowledge about health and illness in ethnic groups in Denmark and about ethnic Danes living in deprived neighbourhoods. The purpose of this study is to investigate associations between self-rated health and ethnicity and social position in a deprived neighbourhood in Denmark in which a relatively largely proportion of the residents are immigrants.

**Methods:**

This study investigates the association between self-rated health used as dependent variable and ethnicity and social position (defined as index for life resources) as the independent variables. The analyses are based on data collected in a survey in a geographically bounded and social deprived neighbourhood, Korskaerparken, located in the municipality of Fredericia in Denmark. The sample consisted of 31% of the residents in Korskaerparken and of these 29% have an ethnic background other than Danish.

The analyses were conducted using logistic regression adjusting for confounding variables.

**Results:**

This study indicates no significant association between ethnicity and having poor/very poor self-rated health.

On the other hand the study confirms that a strong and significant association between the number of residents' life resources and their self-rated health does indeed exist. The results clearly suggest that the more life resources an individual has, the lower is the risk of that individual reporting poor health.

**Conclusion:**

The results show a strong association between the residents' number of life resources and their self-rated health. In this study, we were not able to identify any association between ethnicity and self-rated health, i.e. our results suggest that ethnicity does not constitute an explanation to differences in self- rated health.

## Background

It is well known that health has a social gradient in the sense that people with low socio-economic status (SES) have more health related problems than people with high SES [1,2]. Extensive research into socioeconomic inequalities in health shows that people with high SES live longer and are healthier than people with low SES, who tend to die younger and suffer more illnesses and disabilities [3-6].

In recent years the close connection between SES and differences in health between ethnic groups have been subject to growing interest among researchers [7], and some studies have found an association between ethnicity and long term illness and poor health [8,9]. Denmark has, since the beginning of the 20^th ^century, received immigrants from more than 200 different countries. Approximately half of the immigrants originate from non-western countries such that in 2007 the proportion of adult Non-western immigrants (NWIs) constituted 3.3% of the Danish population [10]. Despite the multi-ethnic composition of the Danish population and the fact that many immigrants live in deprived residential areas there is very limited research-based knowledge about health and illness in ethnic groups in Denmark [11-14] and about ethnic Danes living in deprived neighbourhoods [15,16].

Contributory causes for the lack of research-based knowledge are that it is commen to leave out NWIs in national health surveys, and that there are often so few NWIs in the samples that attempts to generalize are non productive [10]. Literature on health among ethnic minorities in Denmark typically involves a comparison between the ethnic minorities and the (average) majority of ethnic Danes [11,12,17]. Consequently, most studies are based on the assumption that ethnicity/culture is one of the main reasons for health inequality. Singhammer's findings show that the health status is poorer among NWIs than among ethnic Danes in particular, his study shows that the risk of temporary mental illnesses, such as depression and anxiety, are two to four times higher among NWIs compared to ethnic Danes [11].

Differences in health, in terms of morbidity as well as mortality, across ethnic groups have been documented in studies from the United States [18-20] and the United Kingdom [21,22] and to a lesser extent from Europe.

A number of Scandinavian studies have addressed the association between ethnicity and self-rated health [23-25]. The studies show that ethnicity is associated with poor self-rated health, and that socio-economic status only explains part of the association, while another part of the association is explained by acculturation and discrimination.

Although there exist substantial data material on differences across ethnic groups, the factors underlying the differences - especially the role socioeconomic inequalities might play - remain contested. Many researchers still focus on cultural and/or genetic explanations for the differences [26,27] partly due to lack of good data on socioeconomic position which has impeded the investigations of ethnic inequalities in health. Arguments for and against various indicators of socioeconomic position have been given [28,29], these arguments typically aim to identify the effect of a specific socioeconomic determinant on health while adjusting for one or more other indicators. While this approach may have merits in its own right, it nevertheless overlooks the complex socioeconomic pathways through which health inequalities are produced [7,30].

Rather than studying the merits of one specific socioeconomic indicator, we discuss the use of a resource index to measure social position. We believe that an index can provide a better understanding of the complexity of the effect of social position on health by including more socioeconomic indicators in the same index [31].

We regard social position from a generic resource perspective whereby resource allocation within the population is assumed to influence the individual's living conditions in terms of aiding health, well-being etc. The resource perspective is closely connected to the Nordic welfare model which is centred around resource allocation within the population as a whole and focussing on resource development rather than economic redistribution [32-35]. The resource perspective is based on the idea that an individual is an actively acting being with self-defined goals, who in his or her strive to reach these goals is limited by the resources available [33,36].

Using a resource index for measuring social position has a number of advantages [37,38]. In contrast to traditional categorizations based on occupational positions, the resource index for social position allows individuals outside the labour market to be positioned as well as working individuals. Also, an index can include information on family structure and resources within the family, e.g. economic deprivation, which constitute important aspects with regards to social position. A further advantage is that an index measures social position on a continuum which gives more individual measures than a set of rigid social position categories [39]. And last but not least, individuals are evaluated based on their total amount of non-prioritised resources allowing the possession of one resource to make up for a lack of another.

The purpose of this article is to investigate associations between self-rated health and ethnicity and social position in a deprived neighbourhood in Denmark in which a relatively large proportion of the residents are immigrants.

## Methods

The analyses of this article are based on data collected from the geographically bounded and social deprived neighbourhood Korskærparken, located in the municipality of Fredericia in Denmark. A total of 1842 persons live in the neighbourhood, of these 1321 are adults, 36.7% are immigrants (364 adults and 311 children under the age of 18), 4.7% are unemployed, 34.2% are single parents, and 55.2% have a disposable income of less than the median disposable income in Denmark (DKK 150 000 per year)[40]. Of the 364 adult immigrants 224 (61.5%) receive either social benefits (the majority), incapacity benefits or sickness benefits.

**Figure 1:**
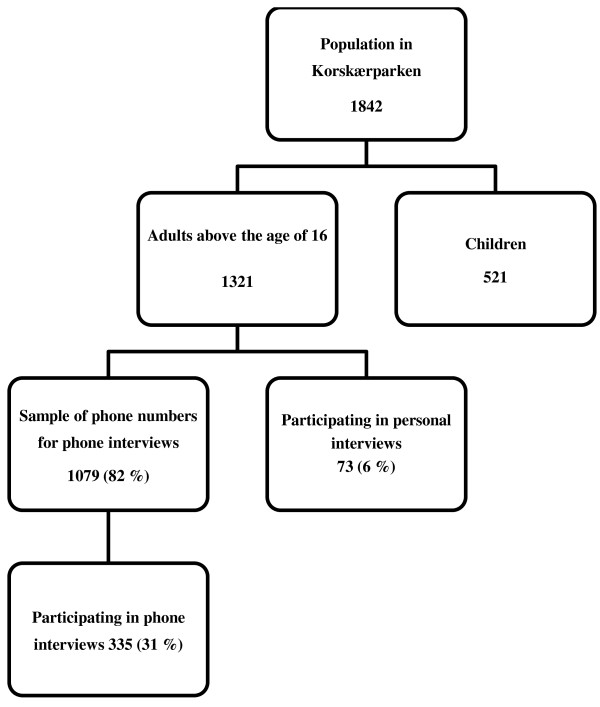
Flow-chart of sampling scheme and interview participation.

The target group was defined as adults above the age of 16 in Korskærparken. The sample consisted of 31% of the residents: 408 agreed to take part in the survey but only 404 answered the questionnaire (Figure 1). Residents with an ethnic background other than Danish made up 29% of the sample, of these seven individuals were from the Nordic countries and further five from the rest of Europe (excluding the former Yugoslavia). It was not registered whether the residents with non-Danish ethnic backgrounds were immigrants or descendents.

The data collection was carried out in the spring of 2008 by the consultancy firm Capacent/Epinion, and the research project is financed by the municipality of Fredericia. Information on the survey was announced in advance throughout the neighbourhood. The sampling was stratified on ethnicity. Data were primarily collected via telephone interviews, and secondary via personal interviews to include those who were not listed in the telephone directory to avoid possible bias. The interviews were carried out by a multilingual team of interviewers with diverse ethnic backgrounds, who could assist with linguistic difficulties related to understanding the questionnaire. It was not registered which of the participants were interviewed by phone and which in person. From 12 households two members were sampled, and from one household three members were sampled. It was not registered which of the participants came from the same households.

### Measures

#### Questionnaire Development

The questionnaire used in the study was constructed in line with existing research [41] and designed to collect information on health indicators, health behaviours, social factors and use of welfare services. The questionnaire, consisting of 50 questions, was developed in connection with an intervention study in Korskærparken running from 2008 to 2011.

#### Self-rated health

Self-rated health is defined as an individual's own view of his or her state of health. Self-rated health is an often used and relatively precise state of health indicator, and is a well-know predictor of mortality and morbidity [42,43]. It can be argued that a negative opinion of one's state of health increases the risk of death, cancer, and heart disorders, which lead to an increased use of the healthcare system and incapacity benefits, more absenteeism, a higher level of medicine use, and reduce the individual's general ability to function, which further lead to a greater risk of unemployment [36].

The questionnaire included the question on self-rated health "How would you assess your present state of health?" (Options: "very good", "good", "fair", "poor", and "very poor"). Due to the small sample size, it was later dichotomized by combining the answers "very good", "good" and "fair" into one category and the answers "poor" and "very poor" into another, as is common in the public health area.

#### Social Position

To measure social position we use an index for life resources based on the index used in a report on self-rated health and inequality in health in the County of Aarhus [36]. The index is a resource index, which in addition to socioeconomic factors e.g. education, job and income also includes family conditions e.g. children and marital status. A similar approach is used in Oakes & Rossi [39], and it is in line with Scandinavian welfare research. The index is based on a formative measurement model where the theoretical variable "social position" is defined by the resource variables included in the index, as opposed to a reflective measurement model where it is assumed that the variables included in the index reflect an underlying theoretical variable (latent variable).

#### Index for life resources

We use a slightly adjusted version of the index by Larsen [36] in that we emphasize economic deprivation and leave out home ownership since all of the homes in the area are rental. The following variables are included in the index for social position: living with others, children, education, occupational income, job category disposable income, and economic deprivation (Table 1).

**Table 1:** Variables applied in the index for life resources

	Resource	Lacking resource
Living with others	Yes	No
Has children	Yes	No
Has studied beyond primary school	Yes	No
Has occupational income	Yes	No
Is white-collar worker or self-employed	Yes	No
Has monthly disposable income ≥ DKK 3 000	Yes	No
Suffers no economic deprivation	Yes	No

The minimum disposable income of DKK 3000 per month after fixed expenses (approx. USD 630/€ 400 in 2008 currency) is the study population median and was used in order to facilitate a comparison between the economicly worse-off and better-off groups of residents in deprived residential areas such as Korskærparken. Economic deprivation is here defined as the situation where a family during the past two months for economic reasons have been unable to pay their bills, cope with incidental expenses, buy birthday presents, afford dental check-ups or buy medicine, clothing, shoes etc.

The index for life resources can attain values from zero to seven. A low index value indicates an individual with few resources a high index value indicates an individual with many resources. We grouped the variable into an ordinal variable with the categories 0-1, 2, 3, 4-7 resources, respectively.

#### Statistical analyses

An exploratory analysis included frequency tables, cross tabulations and chi-square tests.

The relationship between self-rated health and ethnicity was analysed as well as the relationship between self-rated health and the index for life resources. We checked for effect modification between ethnicity and life resources. The analyses were conducted using logistic regression. Age, gender and ethnicity were considered the main relevant confounders and were adjusted for.

The statistical analyses were carried out using SPSS version 17.

## Results

The demographic characteristics of the sample are listed in Table 2.

**Table 2:** Descriptive statistics for the study variables

Variable		N	%
**Gender**	Male	196	48.5
	Female	208	51.5
**Age**	16-24 years	59	14.8
	25-44 years	150	37.6
	45-64 years	109	27.3
	65+	81	20.3
**Ethnicity**	Different ethnic background	118	29.2
	*Europe excluding former Yugoslavia*	*12*	
	*Other*	*106*	
	Ethnic Danes	286	70.8
**Life resources**	0-1	22	8.2
	2	42	15.7
	3	79	29.6
	4-7	124	46.4
**Self-rated health**	Very good/good/fair	343	84.9
	*Very good/good*	*250*	*61.9*
	*Fair*	*93*	*23.0*
	Poor/very poor	61	15.1

About 62% of the participants reported having good/very good health, while 15% reported having poor/very poor health. Around 30% of the participants had a different ethnic background than Danish. The majority of non-Danish residents came from Afghanistan, Iraq, former Yugoslavia, Somalia and Sri Lanka. A small group of 12 came from Europe excluding former Yugoslavia. The sample was almost equally distributed in terms of gender. The age group 25-44 years constitutes the largest group of participants (38%). Effect modification between ethnicity and life resources was not found (p=0.709).

**Table 3:** Risk of having poor/very poor self-rated health for residents of different ethnic background and ethnic Danes

		**Adjusted effect**^**1**^ **OR (95% CI)** **N = 263**	**Effect adjusted for life resources**^**2**^ **OR (95% CI),** **N = 263**
**Ethnicity**			
	Other ethnic background	1.79 (8.89-3.57)	2.03(0.87-4.74)
	Ethnic Danes	Reference	Reference

^1^Model was adjusted for the effect of age and gender

^2^Model was adjusted for the effect of age, gender and life resources

As seen in Table 3, this study indicates no significant association between ethnicity and having poor/very poor self-rated health.

**Table 4:** Risk of having poor/very poor self-rated health in relation to life resources

		**Adjusted effect**^**1**^ OR (95% CI) N = 263	P-value
Life resources	0-1	**13.56 (4.00-45.94)**	**<0.01**
	2	**4.67 (1.63-13.37)**	**<0.01**
	3	**3.48 (1.33-9.08)**	**0.01**
	4-7	Reference	

^1^Model was adjusted for the effect of age, gender and ethnicity

Table 4 shows a strong association between life resources and self-rated health. The results clearly suggest that the more life resources an individual has, the lower is the risk of that individual reporting poor health. The risk of an individual with no or only one life resource reporting poor/very poor health is almost 14 times greater than the risk of an individual with many life resources (4-7). The risk of an individual with 2 or 3 life resources reporting poor/very poor health is 5 and 3 times greater, respectively, than for individuals with many life resources.

## Discussion

The survey carried out in Korskærparken in the municipality of Fredericia has confirmed a strong association between residents' number of life resources and their self-rated health. The risk of a resident with no or only one life resource reporting poor/very poor health is about 14 times greater than the risk of a resident with many life resources (4-7), and the risk of a resident with 2-3 life resources reporting poor/very poor health is 5 and 3 times greater, respectively, than the risk of a resident with many life resources. We were not able to identify an association between ethnicity and self-rated health in Korskærparken. Our analyses suggest that ethnicity and culture are not primary reasons for differences in self-rated health when the study population come from social deprived neighbourhoods. The results we have found in this study are consistent with the results obtained by Larsen [36], even though the study by Larsen differs from our study in certain ways. Our study concerns a socially deprived neighbourhood hosting a relatively large number of residents with few resources, while the study discussed by Larsen concerns the general population in a county. Also, the resource index we use includes slightly different economic variables than Larsen's [36], e.g. disposable income and deprivation. Finally, we tested for the influence of ethnicity, which was not considered in Larsen [36].

### Strengths of the study

The relatively high number of participants with an ethnic background other than Danish constitutes an important feature of the Korskærparken survey in itself, as this section of the population often refrain from taking part in national and regional health surveys. We believe that the high attendance of residents with non-Danish ethnic background in part is a result of the data collection strategy, i.e. a combination of telephone and personal interviews carried out by multilingual interviewers. We believe that giving priority to answers from people with other ethnic background than Danish has a great impact on the results in the analysis. The different data collection methods and more answers from this group could be important in the long run to get a more comprehensive picture of the health state of NWIs in deprived neighbourhoods.

One purpose of our analysis was to investigate whether ethnicity is associated with self-rated health. The data from Korskærparken include a number of comparable social groups in terms of income, education, deprivation and neighbourhood characteristics, unlike other large-scale population studies on health in ethnic minority groups in Denmark, e.g. Singhammer [11]. One reason why Singhammer found large differences between ethnic Danes and ethnic minorities whereas our analyses show no significant differences might be that the large-scale surveys compare ethnic minorities to average ethnic Danes, who have a relatively high number of life resources, instead of comparing them to groups of ethnic Danes with comparable social position and comparable number of life resources.

## Limitations

The strengths of the study are balanced by some methodological limitations. The most important being that the resource-index we use is based on theoretical considerations alone and is not validated. We would need more data from different contexts in order to validate it which further research hopefully will contribute to. The measure of self-rated health is based on a single-item question which might have some drawbacks compared to e.g. SF-36. However, the single-item question is a validated and used measure in epidemiology and social science as a global measure of general health status [44]. Studies show that the single-item measure is a strong and independent predictor of morbidity and mortality [45]. The single-item measure is also used in studies comparing health status between different ethnic groups [46].

Further limitations are connected with the data collection in the survey. In the data collection, it was not registered which of the respondents were interviewed by telephone and which by personal interviews. The type of interview could have an effect on the answers given by the respondents. Among the respondents with non-Danish background, it was not registered whether they were first or later generation immigrants. Again this could have an effect on the answers given. Further, it was not registered who belong to the same household. The clustering could lead to bias and thus affect the results of the analyses. Finally, it was not possible to compare different ethnic groups as there are too few of each nationality to stratify the analysis on ethnicity. With a larger data set it would have been relevant to investigate the heterogeneity between ethnic groups further, in order to analyze differences in health beliefs, health literacy and health behaviour.

It was not an aim of this study to compare the results from Korskærparken with results from a larger study population (e.g. the entire municipality/region), or to results from other more well-off local areas, however, in future research it would be relevant to make this kind of comparisons [47] Also, we would have liked to analyse the differences in gender-effect between various ethnic groups but the sample is too small to stratify on ethnic groups.

## Conclusion

By using a resource index to measure social position in a deprived neighbourhood, we have contributed with new knowledge about the association between self-rated health and social position and ethnicity, respectively. The results show a strong association between residents' number of life resources and their self-rated health. In this study, we were not able to identify any association between ethnicity and self-rated health, i.e. our results suggest that ethnicity does not constitute an explanation to differences in self- rated health.

The results of this research should be considered exploratory on which to base further extended studies. Such studies may consider the effect on self-rated health by various intervening variables such as psycho-social stress, long-term illnesses, health problems and life style (e.g. smoking, exercise).

The results underline the need of further testing and developing an index to measure social position and ethnicity in relation to health.

## Competing interests

The authors declare that they have no competing interests.

## Authors' contributions

PTA, CKB are principal investigators, participated in the design of the study and drafted the manuscript; SV health promotion practitioner for the Korskær project in Municipality of Fredericia; UD and PVL performed the statistical analysis. All authors read and approved the final manuscript.
